# 2265. Immunocompromised Patients, Compromised Guidelines? Practice Patterns of Managing Asymptomatic Bacteriuria in Immunocompromised Patients

**DOI:** 10.1093/ofid/ofad500.1887

**Published:** 2023-11-27

**Authors:** Jacqueline Moore, Daphne-Dominique Villanueva, Logan Stewart

**Affiliations:** West Virginia University, Morgantown, West Virginia; West Virginia University, Morgantown, West Virginia; West Virginia University, Morgantown, West Virginia

## Abstract

**Background:**

Asymptomatic bacteriuria is an issue that plagues the field of medicine. Current guidelines set by the Infectious Diseases Society of America (IDSA) recommend against screening for and treating asymptomatic bacteriuria (ASB) in immunocompromised hosts. However, practice patterns of physicians may deviate from these guidelines. The goal of this study was to gather information on the practice patterns of providers in the fields of transplantation and infectious diseases and determine how closely they follow the recommended approach on managing ASB in immunocompromised hosts.

**Methods:**

We employed the use of a computer-based survey using the REDCap software, and then disseminated the survey to several target groups. The survey was distributed from March 2023 through April 2023. There were 145 total responses. The survey included a scale to assess what participants considered to be diagnostic of urinary tract infection (UTI) (Table 1). The survey presented clinical cases which appeared after a specialty was chosen to ensure each participant was shown cases relevant to their specialty.

**Table 1.**
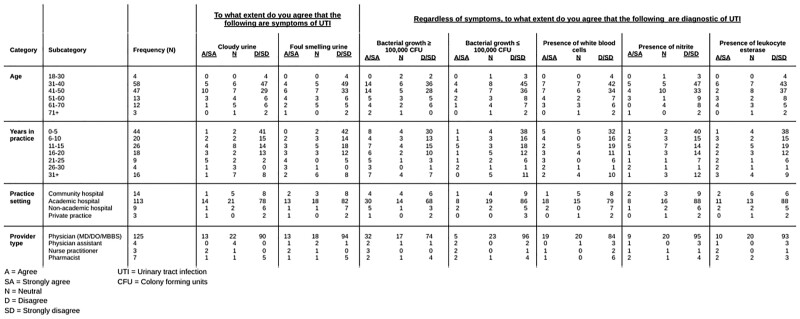
Demographics and Likert scale in diagnosing UTI based on urinalysis components and symptoms

**Results:**

Infectious disease accounted for 111 of the responses. When asked if they would routinely screen for UTI in patients on immunosuppression, 5.8% of participants said yes. Thirty nine participants agreed or strongly agreed that bacterial growth of ≥100,000 CFU on culture is diagnostic of UTI, regardless of symptoms. Seventeen participants saw foul-smelling urine and cloudy urine as symptoms of UTI, 41% of which were infectious disease specialists. A larger number of those practicing more than 10 years versus fewer than 10 years agreed or were neutral regarding foul smelling urine and cloudy urine as symptoms of UTI (Table 1). Figure 1 shows 14.2% of participants chose to treat an asymptomatic neutropenic patient. Of these, 93.8% were infectious disease specialists.

How would you manage a patient with breast cancer on chemotherapy with stable neutropenia who has asymptomatic bacteriuria?

**Figure 1.**
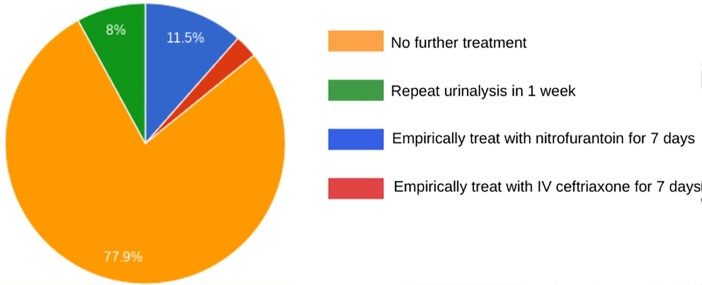
Sixteen out of 114 respondents chose to treat in this clinical case involving an asymptomatic patient with neutropenia

**Conclusion:**

Practice patterns in managing asymptomatic bacteriuria in immunocompromised patients vary, despite latest guidelines. Many providers agreed that foul-smelling urine and cloudy urine are indicators of UTI, and many see bacterial growth as diagnostic. This survey demonstrates room for improvement in management of asymptomatic bacteriuria in immunocompromised patients and the need for further education of providers.

**Disclosures:**

**All Authors**: No reported disclosures

